# Programmable Chiral Radiation via Spin‐Decoupled Metasurface with Integrated Compound Phases

**DOI:** 10.1002/advs.202512669

**Published:** 2025-11-05

**Authors:** Lu Song, Jian Ma, Min Li, Guolong Shi, Zanyang Wang, Xiaofeng Li, Liqiao Jing, Dashuang Liao, Zuojia Wang

**Affiliations:** ^1^ Anhui Provincial Engineering Research Center for Agricultural Information Perception and Intelligent Computing, Anhui Provincial Key Laboratory of Smart Agricultural Technology and Equipment Anhui Agricultural University Hefei 210036 China; ^2^ Air and Missile Defend College Air Force Engineering University Xi'an 710051 China; ^3^ Department of Computer Science Anhui Medical University Hefei 230032 China; ^4^ Zhejiang Key Laboratory of Intelligent Electromagnetic Control and Advanced Electronic Integration Zhejiang University Hangzhou 310027 China; ^5^ Zhejiang Key Laboratory of Intelligent Electromagnetic Control and Advanced Electronic Integration, College of Information Science and Electronic Engineering College of Information Science and Electronic Engineering Zhejiang University Hangzhou 310027 China

**Keywords:** chiral radiation, geometric phase, propagation phase, reconfigurable metasurfaces

## Abstract

Chiral radiation holds promise for applications in sensing, communications, and information processing. Recent advances in optical materials and metasurfaces have enabled unprecedented control over their spectral and momentum characteristics. However, the dynamic reconfigurability of high‐purity chiral radiation remains challenging due to the static architectures and intrinsic spin coupling which degrades polarization purity. Here, a programmable spin‐decoupled metasurface is presented that integrates propagation and geometric phase at deeply subwavelength scales, enabling the generation of high‐purity chiral beams with reconfigurable emission direction. By leveraging chiral radiators with a compound phase‐decoupling strategy, directional control of the desired spin is achieved, whereas the unwanted component is suppressed through circular dichroism‐based amplitude discrimination and phase modulation‐induced destructive interference. Furthermore, the incorporation of positive intrinsic negative (PIN) diodes into the radiators enables active phase modulation, allowing real‐time control over radiation direction. To validate the concept, a 1‐bit programmable chiral metasurface is designed and fabricated with an overall thickness of 0.1λ_0_. Measurements confirm that the metasurface realizes chiral radiation over a wide angular range of ±45°, while maintaining high purity, as evidenced by a 3 dB axial ratio (AR) bandwidth of 10.4%. The proposed approach provides a compact and scalable solution for chirality and directionality engineering.

## Graphical abstract

A programmable spin‐decoupled metasurface integrating propagation and geometric phases enables high‐purity chiral radiation with reconfigurable emission direction. Spin‐selective radiators combined with a compound phase decoupling strategy suppress undesired components, while embedded PIN diodes allow real‐time directional control. A 1‐bit prototype demonstrates ±45° scanning with 10.4% 3 dB axial ratio bandwidth, offering a compact platform for chirality engineering.

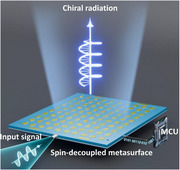

## 1 Introduction

Chirality, defined as the geometric property of an object that cannot be superimposed onto its mirror image, underpins spin‐dependent interactions with circularly polarized light in optics.^[^ ^1^ ^]^ This asymmetry allows emission to favor one handedness over the other, a phenomenon known as chiral radiation or chiral emission,^[^ ^2^ ^]^ which offers a powerful means to generate polarization‐encoded optical signals with broad applications in quantum optics,^[^ ^3^, ^4^ ^]^ sensing,^[^ ^5^, ^6^, ^7^ ^]^ and information processing.^[^ ^8^ ^]^

Various strategies have been pursued to realize chiral radiation, spanning from intrinsic chiral materials to artificially engineered nanostructures. Although natural chiral media, such as optically active crystals,^[^ ^9^ ^]^ can emit circularly polarized light, their weak optical activity and low dissymmetry significantly limit the emission purity and efficiency. In contrast, artificially designed structures, including plasmonics^[^ ^10^, ^11^ ^]^ and metamaterials or metasurfaces^[^ ^12^, ^13^, ^14^, ^15^, ^16^, ^17^ ^]^ with broken symmetry, offer enhanced control over the polarization of light through giant chirality. Particularly, resonant metasurfaces supporting quasi‐bound states in the continuum (quasi‐BIC)^[^ ^18^, ^19^, ^20^, ^21^, ^22^ ^]^ provide a versatile platform for high‐purity chiral emission. By perturbing in‐plane or out‐of‐plane symmetries, chiral metasurfaces enable selective coupling of quasi‐BIC modes to left‐ or right‐handed circular polarizations, producing sharp Fano resonances with near‐unity circular dichroism.^[^ ^23^, ^24^, ^25^, ^26^ ^]^

In addition to polarization control, recent efforts have increasingly focused on manipulating the directionality of chiral radiation.^[^ ^24^, ^26^, ^27^ ^]^ Among these, bilayer metasurfaces enable asymmetric radiation by introducing interlayer symmetry breaking, such as lateral displacement or rotational misalignment, which disrupts both in‐plane and out‐of‐plane mirror symmetries. The combined structural asymmetry facilitates spin‐dependent coupling and directional leakage of quasi‐BIC, resulting in far‐field radiation with controllable polarization and angular asymmetry. However, chiral quasi‐BIC metasurfaces are fundamentally constructed by perturbing symmetry‐protected BICs, where the available states are inherently restricted in momentum space. This intrinsic limitation makes it difficult to steer chiral radiation away from the surface normal or to achieve flexible angular control. Beyond the intrinsic momentum‐space constraints in chiral BIC metasurfaces, introducing geometric phase modulation provides a versatile approach for controlling the directionality of circularly polarized beams,^[^ ^20^, ^28^ ^]^ thus supporting advanced functionalities such as spin‐controlled beam steering and polarization‐multiplexed wavefront shaping.

Despite recent advances, chiral radiation still faces two critical challenges for practical applications: improving polarization purity and achieving dynamic control of the emission direction. Traditionally, generating chiral radiation with a desired polarization has relied on chiral structures that attenuate unwanted circular components through amplitude control, such as spin‐selective absorption^[^ ^28^ ^]^or spin‐selective coupling channels,^[^ ^18^, ^19^, ^21^, ^22^, ^25^ ^]^ while the phase degree of freedom has remained largely untapped. Compound‐phase strategies,^[^ ^29^, ^30^, ^31^, ^32^ ^]^ which exploit both propagation and geometric phases to separate the modulation paths of orthogonal spin states in reflection or transmission, can decouple left‐ and right‐handed spins and have enabled applications such as spin‐multiplexed holography, spin–orbit angular momentum conversion, and space‐multiplexed communications. However, they primarily focus on independent spin control rather than on suppressing the undesired spin component.^[^ ^29^, ^33^, ^34^, ^35^ ^]^ Emerging radiation‐type metasurfaces offer direct wavefront control through programmable meta‐atoms that function simultaneously as radiators and modulators, providing a compact and intrinsically reconfigurable platform for the direct emission of spin‐resolved beams.^[^ ^36^, ^37^, ^38^, ^39^ ^]^ By integrating active components such as PIN diodes or varactors, these metasurfaces enable dynamic phase modulation at the subwavelength level, thereby offering a highly reconfigurable approach to control both chirality and radiation direction in real time.

Here, we propose a programmable spin‐decoupled radiation‐type metasurface that integrates both propagation and geometric phases at a deeply subwavelength scale, enabling reconfigurable generation of high‐purity chiral beams. By leveraging a compound‐phase decoupling strategy on spin‐selective elements that intrinsically support predefined circular polarization, the metasurface allows directional control of the desired spin while suppressing the undesired component via destructive interference, thereby significantly enhancing polarization purity. The incorporation of PIN diodes into the radiating elements enables active phase modulation with 1‐bit resolution, allowing real‐time control over the direction of chiral radiation. To suppress grating lobes caused by 1‐bit phase quantization, a tailored spatial illumination prephase is introduced to the left‐handed circularly polarized (LHCP) component, effectively reshaping the radiation pattern. Simultaneously, a chessboard‐configured prephase is applied to the right‐handed circularly polarized (RHCP) component, diffusing its far‐field pattern and minimizing its contribution, thereby enhancing the purity and bandwidth of the chiral radiation. A prototype metasurface with 8 × 8 meta‐atoms is designed and experimentally validated, demonstrating chiral radiation over a wide angular range of ±45°, a maximum aperture efficiency of 32%, and a 3 dB axial ratio bandwidth of 10.4%. The proposed methodology establishes a new framework for designing low‐profile and reconfigurable chiral radiators, with promising applications in wireless communication, remote sensing, and electronic countermeasure systems.

## 2 Results and Discussion

**Figure 1:**
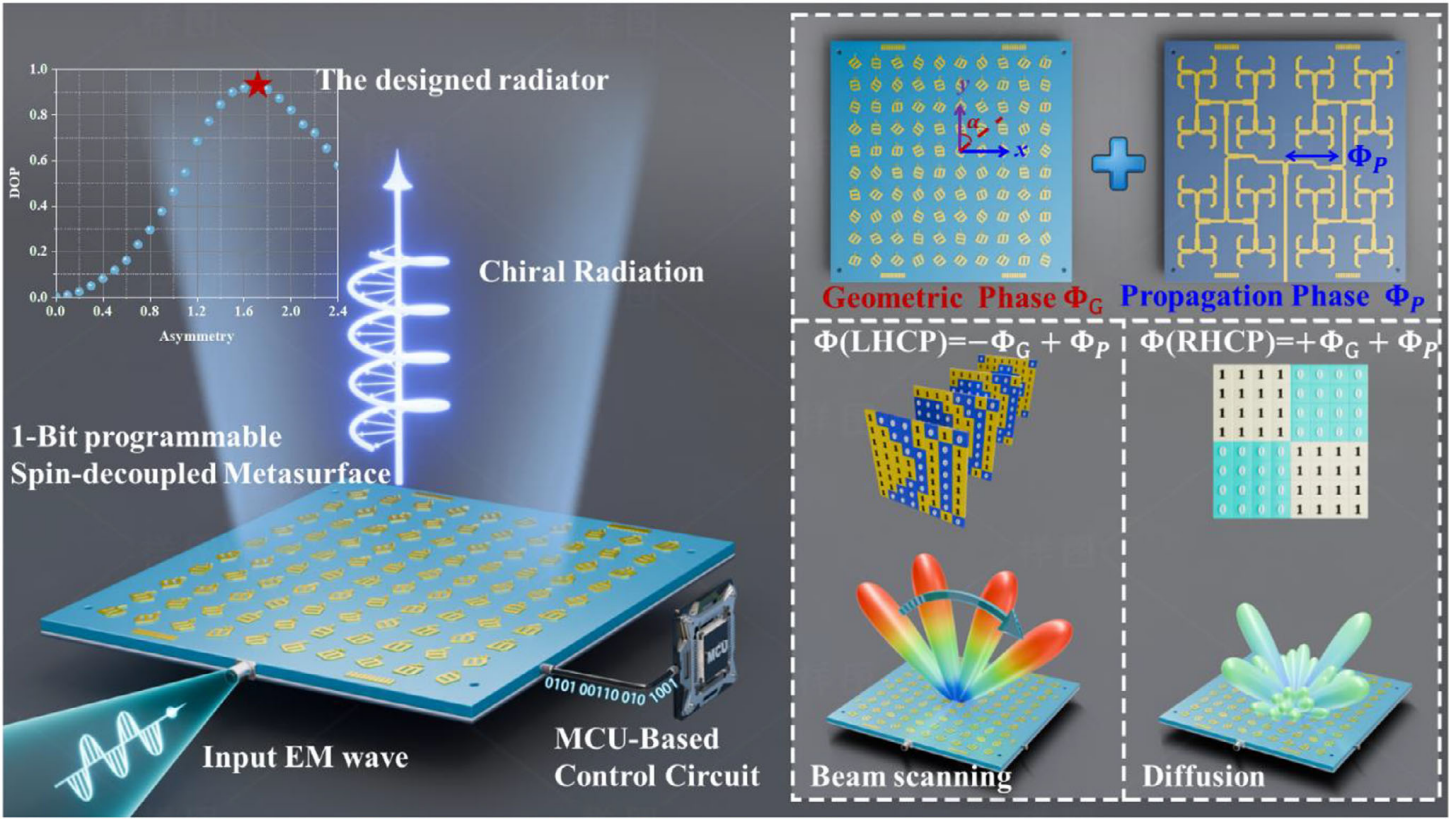
Schematic of the proposed programmable spin‐decoupled metasurface with integrated geometric and propagation phases. The PSDM is composed of periodically arranged meta‐atoms integrated with two PIN diodes each, enabling dynamic binary phase control through multi‐channel bias voltages provided by an MCU‐based control circuit. The spin‐selective meta‐atoms that intrinsically support LHCP, together with a compound phase decoupling strategy, allow directional control of the desired LHCP while suppressing the undesired RHCP component via destructive interference, thereby significantly enhancing polarization purity. Moreover, 1‐bit dynamic phase encoding enables wide‐angle beam steering with low hardware complexity.

The schematic diagram and operational principle of the proposed programmable spin‐decoupled metasurface (PSDM) are illustrated in **Figure** **1**. The top layer consists of periodically arranged meta‐atoms for electromagnetic wave radiation, each incorporating two PIN diodes. To achieve spin‐selective radiation, opposite corners of the square patch are truncated, which deliberately breaks the structural symmetry and induces a clockwise rotation of the surface current (see Note 1, Supporting Information). The degree of asymmetry is characterized by the truncation size, which serves as an additional degree of freedom to control the degree of polarization (DOP), defined as DOP = $\left(r_{LHCP}^{2}-r_{RHCP}^{2}\right)/\left(r_{LHCP}^{2}+r_{RHCP}^{2}\right)$, where *r_LHCP_* and *r_RHCP_* denote the radiation magnitudes of the LHCP and RHCP components, respectively. The relationship between the truncation size and the DOP is illustrated in the inset of Figure 1, the red pentagram indicates the optimized radiator that achieves the best DOP. Based on the above spin‐selective radiators, by combining a compound‐phase decoupling strategy, the metasurface allows directional control of the desired spin while suppressing the undesired component via destructive interference, thereby significantly improving the purity of chiral radiation. A microcontroller unit (MCU) controlled circuit is introduced to provide multiple control voltages, making each meta‐atom individually addressable to achieve dynamically binary phase switching between 0° and 180°. The bottom layer integrates a feeding network, where the signal is injected at the port and propagates as a traveling wave along the branches of the integrated series‐parallel hybrid microstrip network, directly exciting the corresponding meta‐atoms through metallic vias. Beyond serving as a radiator, the meta‐atom also plays a part in introducing the geometric phase to the emitted wavefront through geometric rotation, while the propagation phase is dictated by variations in the feeding line length. The prephase distributions of LHCP and RHCP waves are tailored by joint modulation of geometric phase and propagation phase, respectively, resulting in broadband high‐purity chiral radiation. Furthermore, dynamic binary phase encoding is implemented through PIN diode switching, facilitating wide‐angle beam scanning with low complexity.

### 2.1 Principle of Phase Modulation

We get started from the underlying physics of spin‐decoupled phase modulation. For a meta‐atom positioned in the *xoy* coordinate system, the transmissive response in terms of the circular‐polarization basis can be described using the following transmission matrix,

(1) $$T_{21}^{\mathrm{cir}}=\left[\begin{array}{c} T_{21}\left(\mathrm{LHCP}\right)\\ T_{21}\left(\mathrm{RHCP}\right) \end{array}\right]=\left[\begin{array}{c} A_{L}e^{j\varphi_{L}}\\ A_{R}e^{j\varphi_{R}} \end{array}\right]$$

where *T*_21_(LHCP)/*T*_21_(RHCP) is the transmission coefficient of LHCP/RHCP waves, *A_L_*/*A_R_* denote the amplitude of *T*_21_(LHCP)/*T*_21_(RHCP), and φ_*L*_/φ_*R*_ represent the phase of *T*_21_(LHCP)/*T*_21_(RHCP), respectively.

To facilitate the analysis, the transmission matrix $\;T_{21}^{\;cir}$ can be transformed from the circular basis to the Cartesian coordinate by using a transformation matrix of ***Λ*** =$\;\frac{1}{\sqrt{2}}\left[\begin{array}{cc} 1 & 1\\ -j & j \end{array}\right]$,

(2) $$\;T_{21}^{\;lin}=\Lambda \cdot T_{21}^{\;cir}$$

Since the geometric phase arises from identical element patterns with varying geometric orientations, it can be effectively characterized using a standard rotation matrix.

(3) $$\mathrm{Rot}=\left[\begin{array}{cc} cos\alpha & sin\alpha \\ -sin\alpha & cos\alpha \end{array}\right]$$

where 𝛼 denotes the counterclockwise rotation angle of the top meta‐atom around the +z axis, the transmission coefficient matrix after rotation ***T*** ^′^ can be written as

(4) $$T^{\prime }=Rot\cdot \Lambda \cdot T_{21}^{\;cir}$$

Equation (4) is transformed back into the circular‐polarization basis by applying the inverse matrix of Λ,

(5) $$T\left(\mathrm{Rot}\right)=\;\Lambda^{-1}\cdot \mathrm{Rot}\cdot \Lambda \cdot T_{21}^{\;\mathrm{cir}}=\left[\begin{array}{c} A_{L}e^{j(\varphi_{L}-\alpha )}\\ A_{R}e^{j(\varphi_{R}+\alpha )} \end{array}\right]$$

As dictated by Equations (1) and (5), the rotation of the meta‐atom by an angle 𝛼 induces additional transmission phase shifts of −*α* and +𝛼 for the LHCP and RHCP waves, respectively, inherently manifesting the conjugated symmetry characteristic of the geometric phase. A propagation phase Φ_*P*_ is introduced to operate synergistically with the geometric phase to decouple their conjugate responses. Generally, the phase profile of LHCP and RHCP waves can be expressed as

(6) $$\left\{\begin{array}{c} \Phi_{\mathrm{Ini}}\left(\mathrm{LHCP}\right)=-\alpha +\Phi_{P}\\ \Phi_{\mathrm{Ini}}\left(\mathrm{RHCP}\right)=+\alpha +\Phi_{P} \end{array}\right.$$

Therefore, the rotation angle α and propagation phase Φ_*P*_ can be calculated as

(7) $$\left\{\begin{array}{c} \alpha =\frac{\Phi_{\mathrm{Ini}}\left(\mathrm{RHCP}\right)\;-\;\Phi_{\mathrm{Ini}}\left(\mathrm{LHCP}\right)}{2}\\ \Phi_{P}=\frac{\Phi_{\mathrm{Ini}}\left(\mathrm{LHCP}\right)+\Phi_{\mathrm{Ini}}\left(\mathrm{RHCP}\right)}{2} \end{array}\right.$$

It is noted that the geometric phase arises from element rotation and the propagation phase is determined by the feedline length are fundamentally independent, offering a feasible solution for achieving decoupling wavefronts under different polarization states.

### 2.2 Meta‐Atom Design and Operation Principle

**Figure 2:**
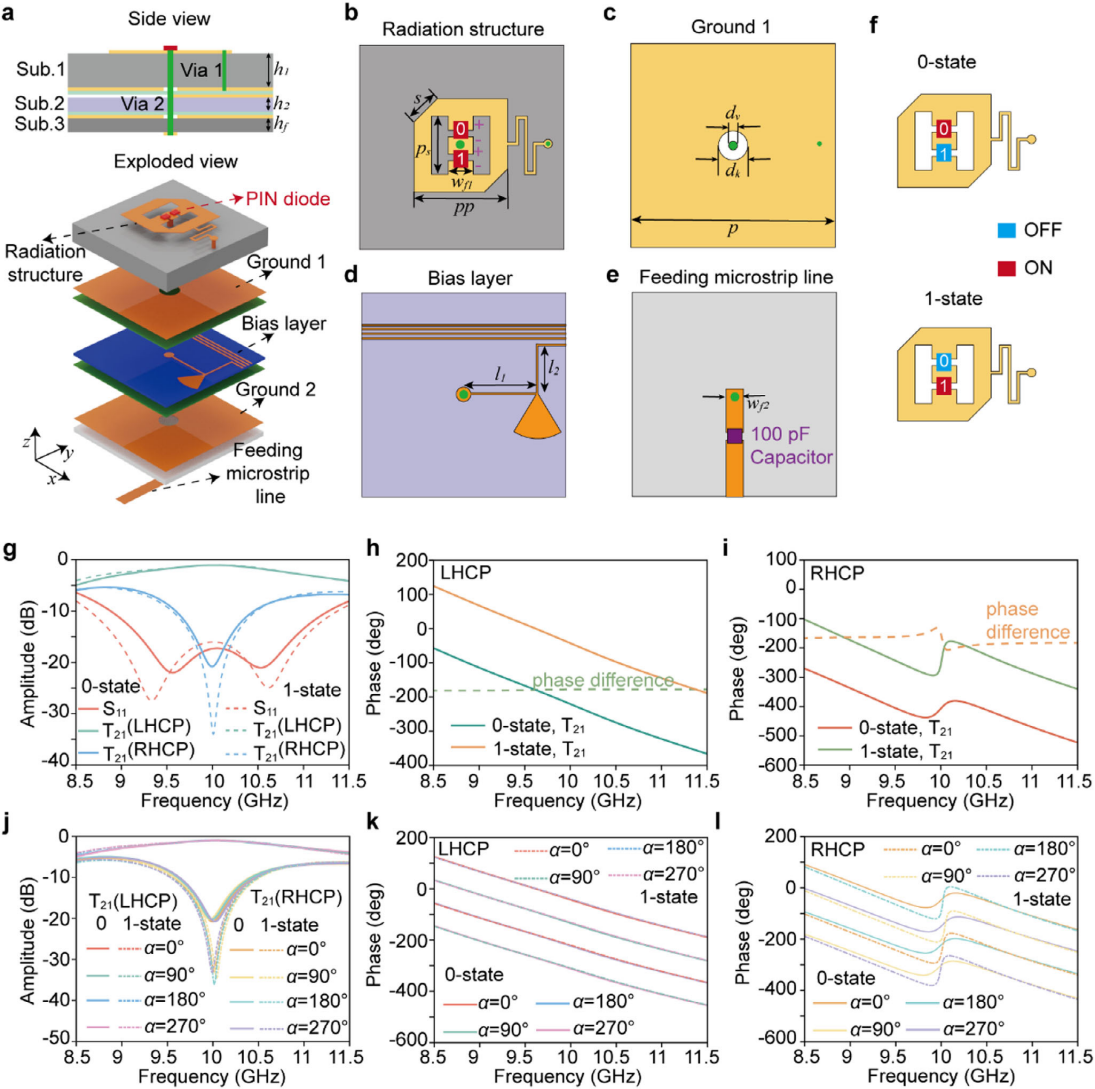
Performance characterization of the meta‐atom. a) Exploded view of the meta‐atom, composed of five metal layers supported by three F4B substrates and two bonding films. b) Top‐layer radiating structure integrating a rectangular patch and a diagonally truncated square patch interconnected by two PIN diodes. c) Ground 1 and metallized via. d) Bias control layer designed with ultranarrow microstrip lines and open radial stubs for high‐frequency signal suppression. e) Feeding microstrip line embedded with a 100‐pF DC blocking capacitor for isolation. f) 0‐state and 1‐state configurations for the radiation‐type meta‐atom. g) Simulated S parameters and transmission magnitudes for LHCP and RHCP components. h) Simulated phase difference of the LHCP transmission coefficient (T_21_) between the 0‐state and 1‐state, showing near‐constant −180° phase shift across 8.5–11.5 GHz. i) Simulated phase difference of the RHCP transmission coefficient (T_21_) for both states, with a sharp phase transition near 9.95 GHz. j) Transmission coefficient (T_21_) of LHCP and RHCP components in the 0‐state and 1‐state. k) Phase of the LHCP component for 0‐state and 1‐state with different rotation angles of α. l) Phase of the RHCP component for 0‐state and 1‐state with different rotation angles of α.

In this section we will discuss the details of implementation and operation principle of reconfigurable spin‐decoupled metasurface. The proposed programmable chiral radiation‐type meta‐atom with 1‐bit phase resolution comprises five metal layers, structurally supported by three F4B substrates and two bonding films, as illustrated in **Figure** **2**a. From top to bottom, these metal layers include the radiating structure, the first ground plane (Ground 1), the bias layer, the second ground plane (Ground 2), and the feeding microstrip line. As depicted in Figure 2(b), the radiating structure consists of a centrally positioned rectangular patch and a square patch with two diagonally truncated corners, electrically interconnected by two PIN diodes (MACOM MADP‐000907‐14020) at the junctions. The anode of PIN 0 and the cathode of PIN 1 are linked to Ground 1 via a quarter‐wavelength transformer and a metallized via (Via 1) as shown in Figure 2c, the cathode of PIN 0 and the anode of PIN 1 are connected to the DC bias circuit through the metallized via 2 located in the center of the meta‐atom. The radiating structure and Ground 1 are printed on the top and bottom surfaces of the first F4B substrate (dielectric constant *ε*_r_ of 3.5, loss tangent *tan*δ of 0.001) with a thickness *h_1_* of 2 mm, respectively. The bias layer, shown in Figure 2d, is meticulously designed with an ultranarrow linewidth and positioned in close proximity to Ground 1 for DC voltage control. It incorporates an additional quarter‐wavelength transformer and an open radial stub for choking the high‐frequency signals. The bias layer is printed on the top of the second F4B substrate (dielectric constant *ε*_r_ of 2.2, loss tangent *tan*δ of 0.001) with a thickness *h_2_* of 0.2 mm. As presented in Figure 2e, a 100‐pF capacitor is embedded in the feeding microstrip line to isolate the DC control signal from other meta‐atoms. Ground 2 and the feeding networks are placed on the opposite side of the third F4B substrate (dielectric constant *ε*_r_ of 2.65, loss tangent *tan*δ of 0.001) with a thickness *h_f_* of 0.5 mm. A metallized via (Via 2) with a diameter of 0.8 mm is employed to connect the feeding microstrip line, bias layer, and radiating structure for signal transfer. Three substrates are bonded using two prepregs (dielectric constant *ε*_r_ of 4 and loss tangent *tan*δ of 0.02) with a thickness of 0.1 mm, resulting in a total thickness of 2.9 mm, equivalent to 0.1λ_0_ at 10 GHz. The optimized structural parameters are *h*_1_ = 2 mm, *h*_2_ = 0.2 mm, *h_f_* = 0.5 mm, *s* = 1.7 mm, *ps* = 3.7 mm, *w_f1_* = 1.4 mm, *pp* = 6.6 mm, *p* = 13 mm, *d_k_* = 2.4 mm, *d_v_* = 0.8 mm, *l*_1_ = 3.5 mm, *l*_2_ = 3.1 mm, and *w_f_*_2_ = 1.3 mm.

Full‐wave simulations of the meta‐atom are performed using the commercial software package CST Microwave Studio, the detailed simulation setup can be seen in Supplementary Information Note 1 (Supporting Information). The 1‐bit phase resolution of the proposed radiation‐type meta‐atom is achieved by inverting the surface current through switching the states of two anti‐symmetrically configured PIN diodes embedded in the radiating layer as illustrated in Figure 2f. The 0‐state is realized by applying a 2.5 V DC voltage to the metal ground 1 while setting the biasing line to 0 V, under which PIN diode 0 is turned ON and PIN diode 1 remains OFF. Conversely, when the biasing line is set to 5 V, PIN diode 0 is switched OFF and PIN diode 1 is switched ON, defining the 1‐state. The simulated S parameters and transmission coefficients of the proposed meta‐atom are shown in Figure 2g, the amplitude responses for both the 0‐state and 1‐state remain nearly identical within the frequency range of 8.5–11.5 GHz. The simulated impedance bandwidths, defined by |S_11_|<−10 dB, are 8.85–11.25 GHz for the 0‐state and 8.65–11.35 GHz for the 1‐state, corresponding to relative bandwidths of 23.9% and 27.0%, respectively. The transmission magnitudes T_21_(LHCP) at the center frequency of 10 GHz for the 0‐state and 1‐state are −1 and −0.9 dB, respectively, indicating that the incident energy is efficiently converted into left‐hand circularly polarized radiation. As shown in Figure 2h, the phase difference of T_21_(LHCP) between the 0‐state and 1‐state is maintained close to −180° across the entire 8.5–11.5 GHz frequency band. Meanwhile, the phase difference of T_21_(RHCP) exhibits a sharp transition around 9.95 GHz but overall remains around −180° within the same frequency range as depicted in Figure 2i. The wideband 180° phase difference observed between the 0‐state and 1‐state is attributed to the symmetric design of the radiating structures. Specifically, the quarter‐wavelength transformer embedded in the radiating layer is engineered to exert minimal influence on the radiation characteristics. As a result, the 0‐state configuration can be regarded as a 180° rotation of the 1‐state structure. This structural symmetry enables the generation of transmission coefficients with nearly identical amplitudes but opposite phase responses across a broad frequency range. These results confirm that the proposed meta‐atom enables 1‐bit radiation phase reconfiguration for both the LHCP and RHCP components. The amplitude and phase responses of T_21_ as functions of the rotation angle α under normal incidence are demonstrated in Figure 2j–l, in which the transmission magnitudes remain essentially unchanged and the phases cover a full 2π range for both LHCP and RHCP components as the radiating structure is rotated from 0 to 360° with a step of 90°. In addition, since each unit requires an individual bias line routed to the feeding edge, the full‐wave simulations are conducted to evaluate the 0‐state and 1‐state radiation performances of the meta‐atom under different numbers of integrated bias lines, as illustrated in Figure S4 (Supporting Information).

### 2.3 Simulated and Measured Results

To validate the capabilities of the proposed meta‐atom design, a metasurface prototype consisting of 8 × 8 unit cells is designed with an aperture size of 104 × 104 mm^2^, and the central frequency is set at 10 GHz. Due to the coarse phase quantization inherent in 1‐bit coding metasurfaces, the presence of grating lobes becomes a critical issue that compromises far‐field beam integrity and radiation efficiency. The quantitative characterization of this phenomenon is detailed in Note 3 (Supporting Information). To alleviate the adverse effects of grating lobes, a phase compensation technique based on the virtual focus approach^[^ ^40^ ^]^ is adopted. This method introduces a spatially varying initial phase distribution across the aperture, emulating a spherical wavefront radiating from a virtual focal point, thereby improving beam quality and suppressing side lobes. The phase distribution of LHCP waves can be calculated as

(8) $$\Phi_{\mathrm{Ini}}\left(\mathrm{LHCP}\right)=-\frac{2\pi }{\lambda_{0}}.\left(\sqrt{{\left(\mathrm{mp}\right)}^{2}+{\left(\mathrm{np}\right)}^{2}+F^{2}}-F\right).$$

where λ_0_ is the free space wavelength at the design frequency, (*m, n*) represent the spatial indices of the meta‐atom within the metasurface, and *p* denotes the period of the meta‐atom. F is the virtual focal distance, which is optimized to 60 mm for better radiation performance. To steer the radiation beam toward a specified direction, a compensation phase is introduced to each meta‐atom, the required phase profile can be calculated as follows

(9) $$\Phi_{\mathrm{scan}}=\frac{2\pi }{\lambda_{0}}p\sin\theta_{0}\left[\left(m\;-\;\frac{1}{2}\right)\cos\varphi_{0}+\left(n\;-\;\frac{1}{2}\right)\sin\varphi_{0}\right]$$

where (θ_0_, φ_0_) denotes the target elevation and azimuth angles, and *p* is the lattice period along both in‐plane axes. The total compensation phase Φ_*Tot*_ can be obtained as

(10) $$\Phi_{\mathrm{Tot}}=-\Phi_{Ini}(\mathrm{LHCP})\;-\;\Phi_{scan}+\Phi_{0}$$

Φ_0_ is the reference phase, which is optimized for scanning beams. Given the 1‐bit phase resolution of the proposed meta‐atom, the continuous phase distribution Φ_*Tot*_ is further quantized into two discrete states (0 and 𝜋), corresponding to the 0‐state and 1‐state configurations of the meta‐atom.

(11) $$\Phi_{\mathrm{Tot}}^{Q}=\left\{\begin{array}{c} 0,\Phi_{Tot}\in \left[0+2k\pi ,\pi +2k\pi \right)\;\\ \pi ,\;\Phi_{Tot}\in \left[\pi +2k\pi ,2\pi +2k\pi \right)\;\; \end{array}\right.k=0,\pm 1,\pm 2$$

A circularly polarized radiation‐type metasurface enabled solely by geometric phase modulation is designed for comparison as detailed in Note 4 (Supporting Information), due to the inverted initial phase responses of LHCP and RHCP components, the RHCP is also steered to broadside under this configuration, resulting in a narrow axial ratio bandwidth similar to that of a single meta‐atom.

**Figure 3:**
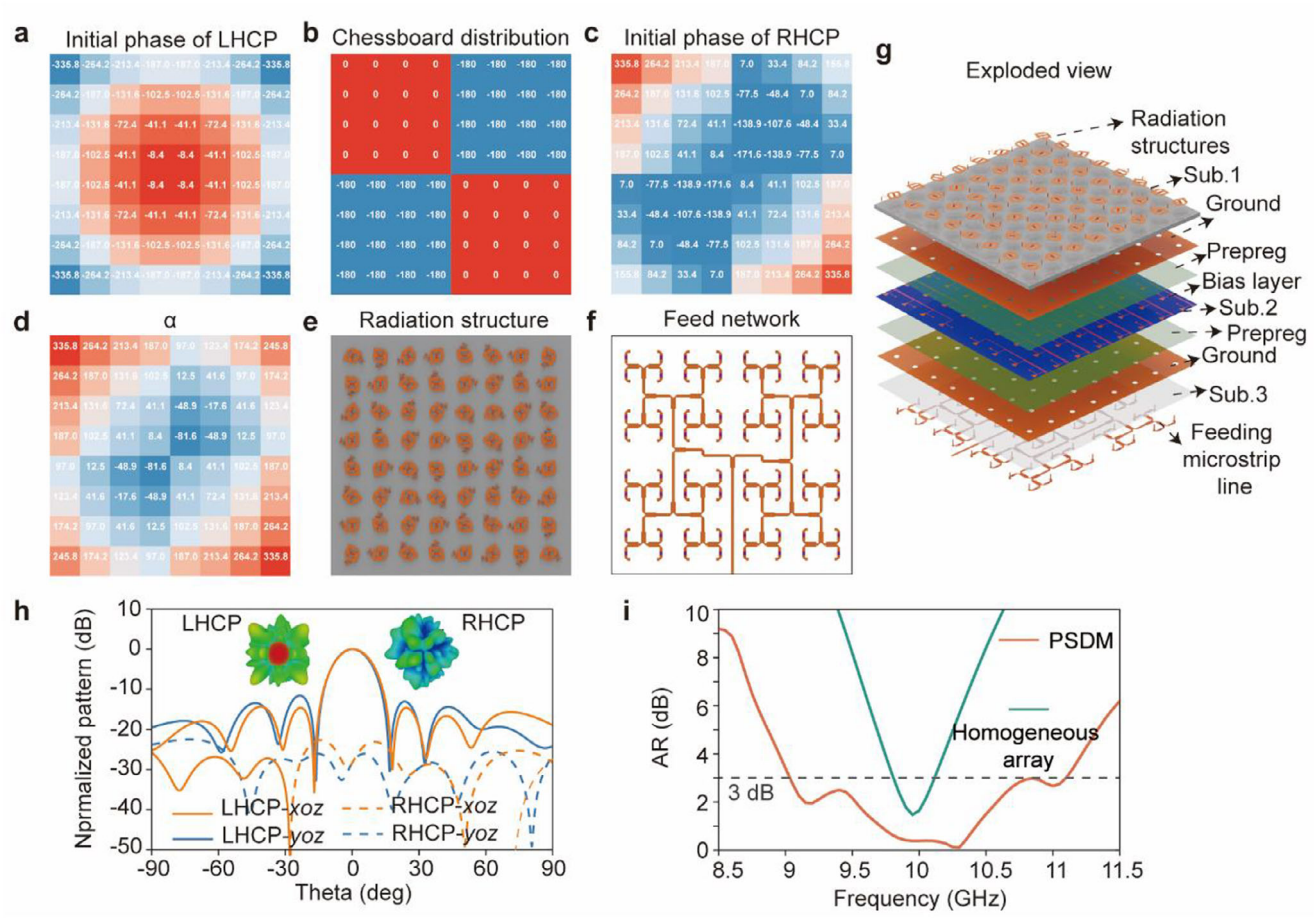
Design strategy and performance enhancement of the PSDM. a) Initial phase distribution of LHCP waves. b) Chessboard‐like phase distribution Φ_*chess*_ used to modulate the RHCP initial phase. c) Initial phase distribution of RHCP waves. d) Distribution of rotation angle α. e) Radiation layer configurations. f) Microstrip feeding network with a hybrid series‐parallel topology, designed to implement the required propagation phase. g) Exploded view of the complete PSDM. h) Simulated radiation patterns in the broadside direction, demonstrating suppression of the RHCP main lobe and redistribution into four side lobes. i) 3 dB AR bandwidth of the homogeneous array and PSDM.

To ensure high purity of chiral radiation, it is essential to suppress or spatially redistribute the RHCP components. This can be achieved by imparting a spatially varying or pseudo‐random phase profile to the initial phase distribution of the RHCP wave Φ_*Ini*_(RHCP), such that the RHCP waves emitted from different meta‐atoms no longer maintain constructive interference in the same direction. Thus, these components experience phase decoherence in the far field. As a result, the RHCP energy is diffused over a wide angular range, minimizing its presence in the main radiation direction. In contrast, the LHCP wavefront remains coherent and focused. This selective diffusion leads to an improved AR in the intended direction of radiation. Thus, the initial phase for the LHCP component Φ_*Ini*_(LHCP) is set as the focusing phase 𝛷_F_ calculated by Equation (8) as shown in **Figure** **3**a. Meanwhile, the initial phase of the RHCP component Φ_*Ini*_(RHCP)is deliberately engineered as Φ_*Ini*_(RHCP) = ‐Φ_*F*_ + Φ_*chess*_, Φ_*chess*_ is a chessboard‐like distribution as presented in Figure 3b, Φ_*Ini*_(RHCP) is illustrated in Figure 3(c). Thus, according to Equation (7), the rotation angle *α* and the propagation phase Φ_*P*_ can be determined as:

(12) $$\left\{\begin{array}{c} \alpha =\;-\Phi_{\mathrm{Ini}}\left(\mathrm{LHCP}\right)+\Phi_{\mathrm{chess}}/2\\ \Phi_{P}=\Phi_{\mathrm{chess}}/2 \end{array}\right.$$

The rotation angles α and the corresponding radiation layer configurations are depicted in Figure 3d,e, respectively. The microstrip feeding network designed according to the required propagation phase Φ_*P*_ is illustrated in Figure 3f, and the detailed design procedure is provided in Note 5 (Supporting Information). It adopts a hybrid series‐parallel topology, where the signal propagates outward from the center through integrated microstrip branches. Equal‐amplitude excitation is ensured by impedance matching, while the desired phase difference is achieved by precisely adjusting the lengths of individual microstrip lines.

An exploded view of the configuration of the PSDM is presented in Figure 3g. The detailed architecture biasing network is provided in Figure S9a (Supporting Information). The full‐wave simulation results for the metasurface radiating in the broadside direction are illustrated in Figure 3h. Compared to Figure S8d (Supporting Information), it is evident that the main lobe of the RHCP component is split into four subsidiary beams, effectively suppressing the RHCP level in the direction of the LHCP main beam. Consequently, the 3 dB AR bandwidth, as shown in Figure 3i, is substantially improved from 9.85–10.1 GHz for a homogeneous array of the same size without any prephase to 9.05–11.05 GHz for the PSDM.

**Figure 4:**
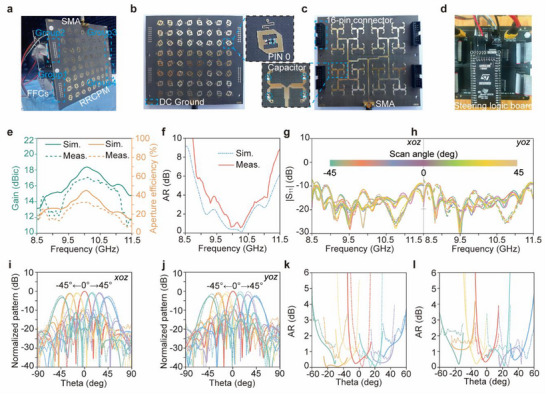
Fabrication, measurement setup, and experimental performance of the prototype PSDM. a) Photograph of the fabricated metasurface prototype and measurement setup inside an anechoic chamber. b) Top view of the metasurface showing 128 PIN diodes divided into four independently controlled groups. c) Backside view of the metasurface with an integrated SMA connector for feeding network excitation. d) Architecture of the steering logic board. e) Simulated and measured realized gain and aperture efficiency of the PSDM from 8.5 to 11.5 GHz. f) Simulated (solid lines) and measured (dashed lines) AR performance in the broadside direction. Measured |S_11_| under different scanning angles in the *xoz*‐plane g) and *yoz*‐plane h) from –45 to +45°. The normalized radiation patterns of scanning beams at i) *xoz*‐plane and j) *yoz*‐plane. Measured AR values versus scanning angles in the *xoz*‐plane k) and *yoz*‐plane l) over 9.6–10.65 GHz.

As illustrated in **Figure** **4**a, a prototype of the PSDM is fabricated and experimentally measured in an anechoic chamber to verify the feasibility of the design. To enable independent manipulation of the transmission states at the pixel level, a 64‐way DC bias circuit is developed to deliver precise control voltages, which necessitates an additional 3 mm extension on both sides of the metasurface for bias line feeding integration. As a result, the overall footprint of the proposed PSDM extends to 110 × 110 mm^2^, while the effective radiating aperture remains confined to 104 × 104 mm^2^. As depicted in Figure 4b, a total of 128 PIN diodes are soldered onto the metasurface and divided into four independently controlled groups via 128 separate bias lines. Power delivery and signal routing are achieved using four flexible flat cables (FFCs) that connect each group to the steering logic board. An SMA connector is integrated onto the backside of the metasurface to excite the microwave feeding network as shown in Figure 4c. The architecture of the steering logic board is shown in Figure 4d, with detailed design procedures provided in Supplement Information Note 6 (Supporting Information).

As shown in Figure 4e, the measured realized gain reaches a peak of 17.1 dBic at 10.1 GHz, which compares favorably with the simulated value of 18.4 dBic. In addition, the measured maximum aperture efficiency is 32% at 10.05 GHz, while the simulated value reaches 45% at 9.95 GHz. It is worth noting that the aperture efficiency remains above 20% across the frequency band from 9.35 to 10.45 GHz. The discrepancies between measured and simulated results are mainly attributed to fabrication tolerances, modeling approximations for the PIN diodes, and measurement setup (e.g., SMA connectors and cable effects are not considered in the simulation). In terms of polarization performance, Figure 4f shows the AR of the broadside beam, with a measured 3 dB AR bandwidth ranging from 9.6 to 10.65 GHz (10.4%). In comparison, simulations indicate a broader bandwidth of 19.9%, extending from 9.05 to 11.05 GHz. The 3 dB AR bandwidth can be strongly influenced by the specific shape and resonance behavior of the S‐parameters. Small deviations in amplitude or phase responses can shift the frequency at which the AR reaches 3 dB, leading to a notable difference between simulation and measurement. Therefore, assessing performance solely based on the 3 dB bandwidth may be insufficient. To provide a more comprehensive evaluation of the AR performance, we compared measured and simulated bandwidths at 1, 2, and 4 dB levels. The simulated (measured) bandwidths are 6.5% (3.9%), 9.95% (7.4%), and 21.8% (18.7%), respectively, showing good agreement across these criteria.

To further evaluate the beam steering capabilities of the PSDM, reflection coefficients |S_11_| are measured under varying scanning angles from –45° to +45° in both the *xoz*‐ and *yoz*‐planes, with 15° increments. As plotted in Figure 4g,h, all configurations maintain a |S_11_| < –10 dB bandwidth from 8.95 to 11.5 GHz, consistent with full‐wave simulations. Normalized radiation patterns of the scanning beams along the xoz‐ and yoz‐planes at 10 GHz are depicted in Figure 4i,j, respectively. The measured patterns show good agreement with the simulated results, exhibiting maximum scanning gain loss of 2.7 dB and 2.2 dB for ±45° beams in the xoz‐ and yoz‐planes, respectively. The code distributions and 3D simulated far‐field radiation patterns for different scanning angles are provided in Supplement Information Note 7 (Supporting Information), and the specific radiation performances of the beam scanning are listed in Table S1 (Supporting Information). ARs as functions of scanning angles in the *xoz*‐ and *yoz*‐planes, depicted in Figure 4k,l, remain below 3 dB for all scanning directions from 9.6 to 10.65 GHz, validating the polarization stability of the proposed design.

A comparative summary of the proposed PSDM and representative chiral radiation metasurfaces is provided in Table 1. It should be emphasized that the proposed PSDM realizes dynamical and high‐purity circularly polarized radiation with spin‐decoupled phase modulation for the first time. Beyond its reconfigurability, the PSDM design also achieves the highest aperture efficiency and the lowest profile among all listed designs. This performance is primarily attributed to the integrated design of the feed and meta‐atom, which effectively eliminates illumination and leakage losses commonly associated with external feeding structures, thereby significantly enhancing the overall integration level of the feed‐metasurface system. In addition, the incorporation of PIN diodes into the spin‐decoupled meta‐atoms enables real‐time steering of chiral beams across a wide angular range of ±45°, while maintaining a 3 dB AR bandwidth of 10.4%.

**Table 1:** Comparison with some representative chiral radiation metasurfaces.

Refs.	[32]	[31]	[29]	[30]	This work
Freq. (GHz)	8.6	11.1	14	27	10.0
Element number	30 × 30	24 × 24	25 × 25	21 × 21	8 × 8
Peak gain (dBi)	NA	NA	21.8	23.2	17.1
Max. aperture efficiency (%)	31.8	NA	26	31.3	**32.0**
AR bandwidth(%)	NA	NA	5	22.3	10.4
Polarization	CP	CP	CP	CP	CP
Method	GP	GP	PP+GP	PP+GP	PP+GP
Profile	5.8λ_0_	74λ_0_	3.8λ_0_	6.2λ_0_	**0.1λ_0_**
Complexity of feeding	Passive	Passive	Passive	Passive	**Active**
Scan range (deg)	−15/+15 (1D)	−30/+30 (1D)	0 (2D)	0 (2D)	**±45** **(2D)**

NA: not available; PP: propagation phase; GP: geometric phase.

## 3 Conclusion

We have presented a programmable spin‐decoupled metasurface capable of dynamically generating high‐purity chiral beams with 1‐bit phase resolution. A generalized design methodology for compound‐phase radiation‐type metasurfaces is developed, enabling decoupling of LHCP and RHCP phase responses. Specifically, a spatially varying illumination prephase is introduced in the LHCP component to suppress grating lobes caused by 1‐bit phase quantization, while a chessboard‐configured prephase is applied to the RHCP component to suppress RHCP contribution and enhance the purity of the LHCP component. A prototype comprising 8×8 elements with a low profile of 0.1*λ_0_* is fabricated and experimentally validated. The measured results verify that the proposed metasurface enables beam scanning over a wide angular range of ±45°, achieves a peak gain of 17.1 dBic at 10.1 GHz, reaches a maximum aperture efficiency of 32% at 10.05 GHz, and maintains a 3 dB axial ratio bandwidth of 10.4% from 9.6 to 10.65 GHz. This work establishes a unified strategy for broadband chiral radiation metasurfaces with high polarization fidelity and reconfigurability, which holds great potential for next‐generation wireless communications, advanced sensing, and multifunctional integrated platforms.^[^ ^41^, ^42^ ^]^ In present work, the achievable propagation phase is constrained by the physical topology of the feeding network, which may limit the realization of arbitrary compound phases. A promising solution is to employ a dual geometric phase design, such as cascading two layers of chiral meta‐units, each providing an independent geometric phase degree of freedom. This strategy enables chiral radiation with independent and arbitrary control for left‐ and right‐handed components^[^ ^43^, ^44^, ^45^, ^46^ ^]^ and will be further explored in our future work.

## Conflict of Interest

The authors declare no conflict of interest.

## Supporting information



Supporting Information

## Data Availability

The data that support the findings of this study are available from the corresponding author upon reasonable request.
