# Supplementary material for: Programmable Chiral Radiation via Spin‐Decoupled Metasurface with Integrated Compound Phases
Source: Adv Sci (Weinh). 2025 Nov 5;13(1):e12669. doi: 10.1002/advs.202512669 (PMC12767010; doi:10.1002/advs.202512669)
Supplement: Supplementary file 1 — Supporting Information [file ADVS-13-e12669-s001.docx]

Supporting Information

Programmable Chiral Radiation via Spin-decoupled Metasurface with Integrated Compound Phases

Lu Song^#^, Jian Ma^#^, Min Li,* Zanyang Wang, Xiaofeng Li*, Liqiao Jing, Dashuang Liao*, and Zuojia Wang

[Note 1 Principle of the meta-atom 2](#_Toc175132597)

[Note 2 Impact of bias lines on the radiation performance of the meta-atom 3](#_Toc175132600)

[Note 3 Analysis of beam-scanning performance with finite phase quantization 4](#_Toc175132600)

[Note 4 Programmable radiation metasurface enabled solely by geometric phase modulation 6](#_Toc175132601)

[Note 5 Overall configuration of the proposed PSDM 7](#_Toc175132602)

[Note 6 Schematic of the steering-logic board 8](#_Toc175132603)

[Note 7 Radiation patterns of the beam scanning 9](#_Toc175132604)

[Note 8 Radiation performances of the beam scanning 10](#_Toc175132605)

**Note 1.** **Principle of the meta-atom**

As illustrated in Fig. S1(a), the proposed reconfigurable chiral radiation-type meta-atom is excited by a waveguide port (Port 1), and periodic boundary conditions and a Floquet port (denoted as Port 2) are employed to numerically analyze its electromagnetic performance.

To realize dynamic control of the transmission phase, two identically oriented PIN diodes are integrated into the radiating structure. The PIN diodes, MACOM MADP-000907-14020, can be modeled as equivalent lumped components during simulation as shown in Fig. S1(b). A series R-L circuit with resistance R = 7.8 Ω and inductance L = 30 pH for positive biasing (ON-state), and as a series C-L circuit with capacitance C = 0.025 pF and inductance L = 30 pH for negative biasing (OFF-state). A lumped inductor with a value of 82 nH is inserted between the circularly polarized receiving antenna and Via 3 for impedance matching and bias isolation. As illustrated in Figs. S1(c) and S2(d), full-wave simulations demonstrate that the proposed meta-atom achieves an impedance-matching bandwidth of 35.2% ranging from 8.65 GHz to 12.35 GHz, defined by |S₁₁| ≤ –10 dB. Meanwhile, the AR remains below 3 dB within a fractional bandwidth of 5.5% (9.75–10.3 GHz), indicating its effective circular polarization performance over this frequency range.


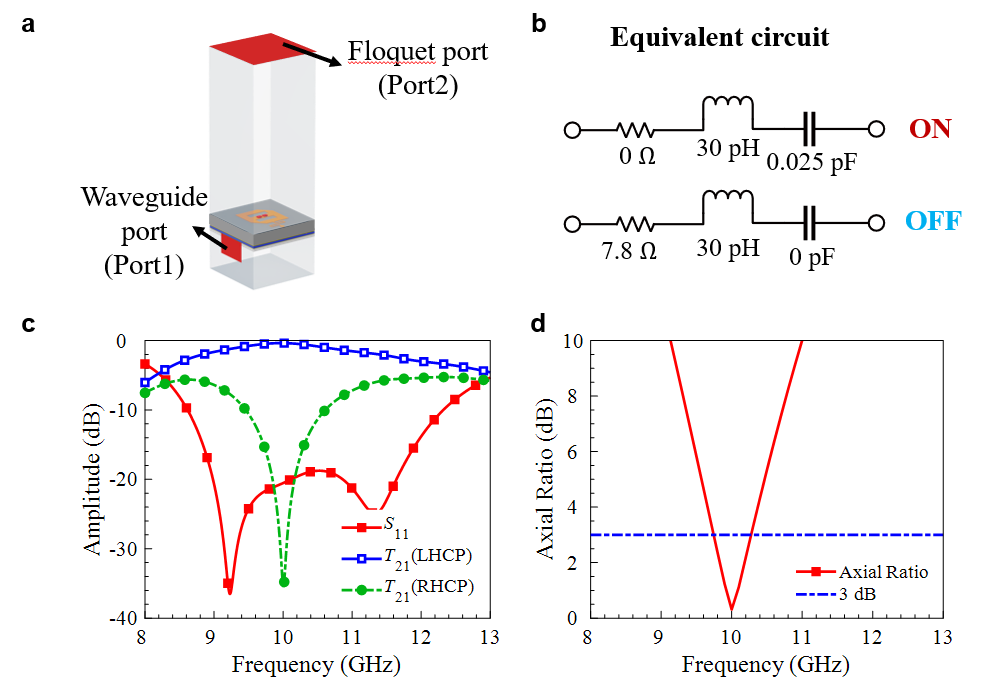


**Figure S1.** (a) Simulation set-up. (b) Equivalent lumped components for PIN diode. (c) S-parameters. (d) AR

In a square patch antenna, the two orthogonal modes (TM10 and TM01) are inherently degenerate, sharing the same resonant frequency. Without perturbation, both modes may be simultaneously excited, leading to circular polarization or unstable polarization states. By introducing a rectangular slot on the patch, the structural symmetry is broken, thereby lifting the mode degeneracy. The slot perturbs the current distribution and alters the effective electrical length in one direction, which shifts the resonant frequency of one mode away from the operating point. As a result, only a single mode dominates at the design frequency, leading to robust linear polarization. Figure R* illustrates the surface current distributions of the meta-atom under different polarization states at the operating frequency of 10 GHz. As shown in Fig. S2(a), introducing a horizontal rectangular slot into the square patch perturbs the current path along the x-axis, thereby generating a dominant horizontal current and exciting linearly polarized radiation in the horizontal direction. Similarly, as presented in Fig. S2(b), a vertical slot enforces the current flow along the y-axis, leading to vertically polarized radiation.

To achieve circular polarization, opposite corners of the patch are truncated, which deliberately breaks the structural symmetry. This perturbation couples the two orthogonal modes (TM10 and TM01) and enforces a ±90° phase difference between them. The superposition of these modes with equal amplitudes and quadrature phase results in circularly polarized radiation. Furthermore, the orientation of the truncations determines whether the phase leads or lags, and thus dictates the handedness of the polarization. Specifically, as shown in Fig. S2(c), truncating the top-left and bottom-right corners induces a clockwise rotation of the surface currents, producing left-handed circular polarization. In contrast, as shown in Fig. S2(d), truncating the top-right and bottom-left corners causes a counterclockwise rotation of the currents, leading to right-handed circular polarization.


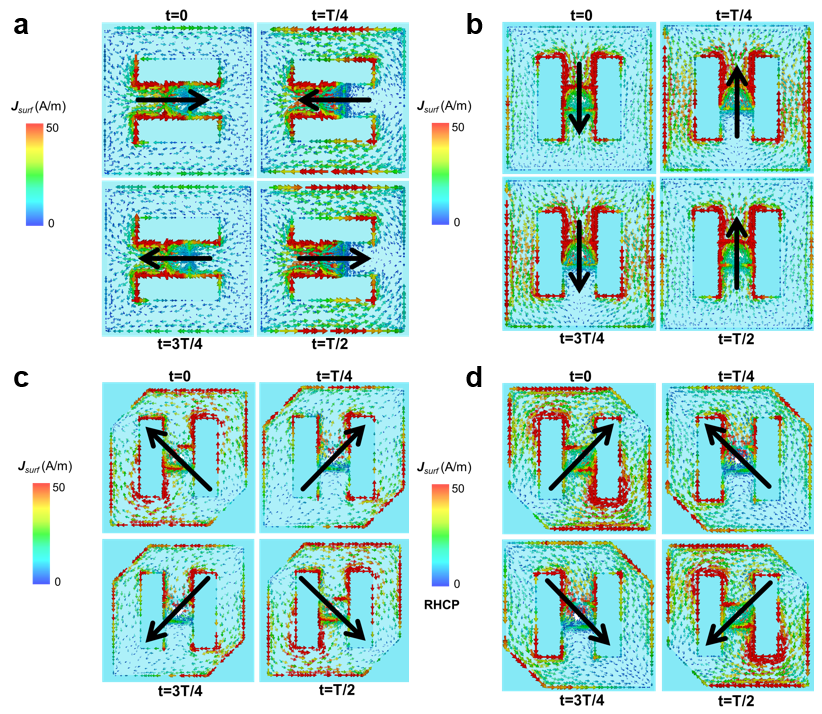


**Figure S2.** Surface current distributions of the meta-atom under different polarization states at the operating frequency of 10 GHz. (a) Horizontal polarization. (b) Vertical polarization. (c) LHCP. (d) RHCP.

To further validate the polarization characteristics, we analyzed the relationship between the corner truncation size and the degree of polarization (DOP), defined as

DOP =$\frac{r_{LHCP}^{2}-r_{RHCP}^{2}}{r_{LHCP}^{2}+r_{RHCP}^{2}}$ (S1)

where $r_{LHCP}$ and $r_{RHCP}$ denote the magnitudes of the LHCP and RHCP components, respectively. The degree of asymmetry is characterized by the length of the truncated corner. Fig. S3(a) shows the magnitudes of the LHCP component as the truncation size increases, while Fig. S3(b) presents the corresponding magnitudes of the RHCP component. It can be observed from Fig. S3(c) that with small or no truncation, the meta-atom exhibits a low DOP due to the dominance of linear polarization. As the asymmetry increases, the DOP gradually improves, reaching its maximum when the two orthogonal modes (TM10 and TM01) are effectively coupled with a 90° phase difference. This indicates the optimal condition for circular polarization. However, further increasing the truncation size disturbs the mode balance, leading to a reduction in DOP. These results confirm that the corner truncation not only breaks the symmetry of the square patch to enable circular polarization, but also provides a controllable parameter to tailor the proportion of chiral radiation components.


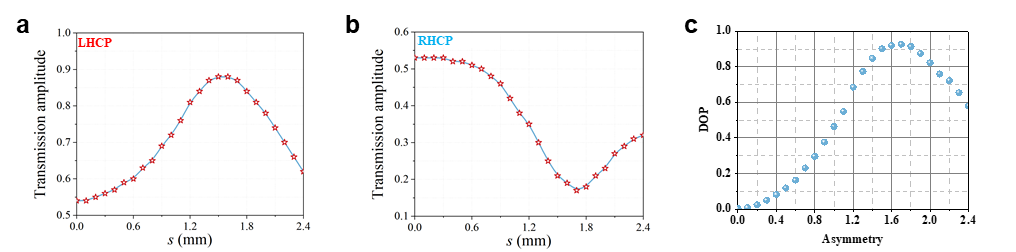


**Figure S3**. (a) Magnitudes of the LHCP component as a function of the truncated corner length. (b) Magnitudes of the RHCP component as a function of the truncated corner length. (c) Relationship between the asymmetry and the degree of polarization.

**Note 2.** **I****mpact of bias lines on the radiation performance of the meta-atom**

To investigate the effect of the number of DC bias lines on the performance of the unit, full-wave simulations are conducted for 0-state and 1-state configurations with varying numbers of integrated bias lines, as illustrated in Figure S4. The results show that increasing the number of bias lines from 1 to 8 has a negligible impact on the magnitude and phase of the transmission coefficient$\text{T}_{\text{21}}\text{(LHCP)}$ and $\text{T}_{\text{21}}\text{(RHCP)}$. Meanwhile, the phase differences between the two states for both $\text{T}_{\text{21}}\text{(LHCP)}$ and $\text{T}_{\text{21}}\text{(RHCP)}\text{ }$remain stable around -180°, indicating that the designed quarter-wavelength transformer and open radial stub effectively isolate the DC bias from the RF signal path.


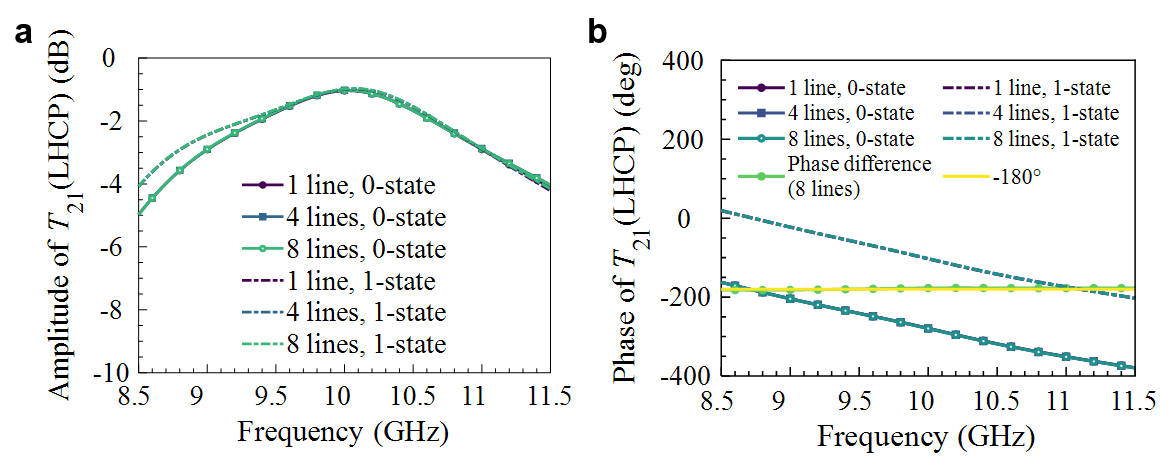


**Figure S4.** Amplitude (a) and phase (b) of $\text{T}_{\text{21}}\text{(LHCP)}$ with different DC bias lines.

**Note 3. Analysis of beam-scanning performance with finite phase quantization**

According to antenna array theory, the far-field radiation pattern of a metasurface can be analytically derived based on the phase and amplitude distribution of its constituent unit cells

$E\left( \theta,\varphi\right)= \sum_{m=1}^{M} \sum_{n=1}^{N} \cdot\left| A_{mn} \right|\cdot e^{jk\phi_{Qmn}}\cdot e^{jku_{mn}}$ (S2)

$u_{mn}=\sin\theta\cdot[(m-\frac{M+1}{2})\cdot p_{x}\cdot cos\varphi+(n-\frac{N+1}{2})\cdot p_{y}\cdot sin\varphi]$ (S3)

where |A*ₘₙ*| denotes the transmission or reflection amplitude of the element, and *p_x_, p_y_* are the lattice constants along *x* and *y* directions, respectively. The indices m ∈ [1, M] and n ∈ [1, N] correspond to the unit cell positions within the M × N array.

To investigate the effect of phase quantization, analytical calculations are performed on metasurfaces with varying quantization levels (1 bit, 2 bit, 3 bit, and continuous phase) and aperture sizes (8×8, 16×16, and 32×32), as presented in Figures S5-S7. It is observed that 1-bit metasurfaces inherently generate symmetric dual beams due to their limited phase resolution. In contrast, metasurfaces with higher phase resolution (≥2-bit) provide greater flexibility in beamforming and beam steering. Specifically, in 2-bit implementations, the beam symmetry is effectively eliminated. However, elevated sidelobes are still present due to residual quantization errors. Further improvement is achieved with 3-bit quantization, where the sidelobe levels are significantly suppressed. Importantly, increasing the aperture size from 8×8 to 32×32 elements enables the far-field pattern to closely approach that of an ideal continuous-phase profile. Nevertheless, simply enlarging the aperture cannot fully compensate for low phase resolution.


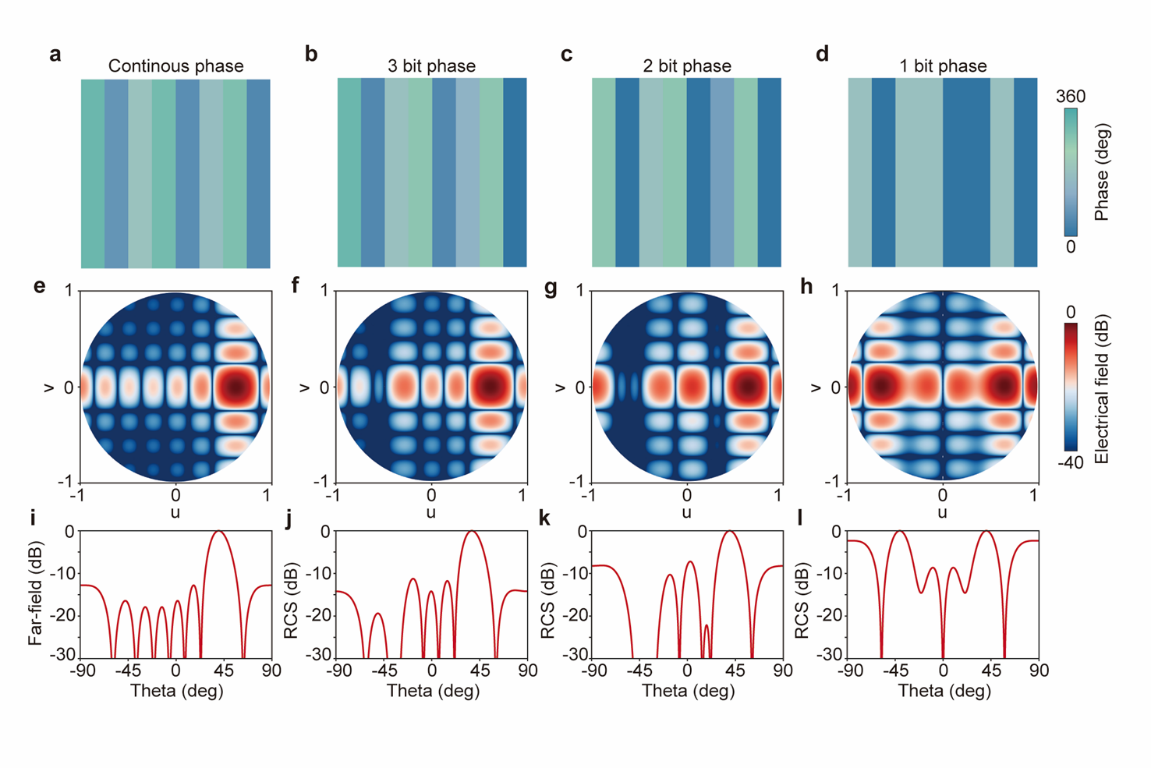


**Figure S5**. 8×8 array steered towards (0°, 45°). Phase distributions for (a) continuous phase, (b) 3-bit quantization, (c) 2-bit quantization, and (d) 1-bit quantization. uv-plane radiation patterns for (e) continuous phase, (f) 3-bit quantization, (g) 2-bit quantization, and (h) 1-bit quantization. Normalized 2D radiation patterns in the φ = 0° plane for (i) continuous phase, (j) 3-bit quantization, (k) 2-bit quantization, and (l) 1-bit quantization.


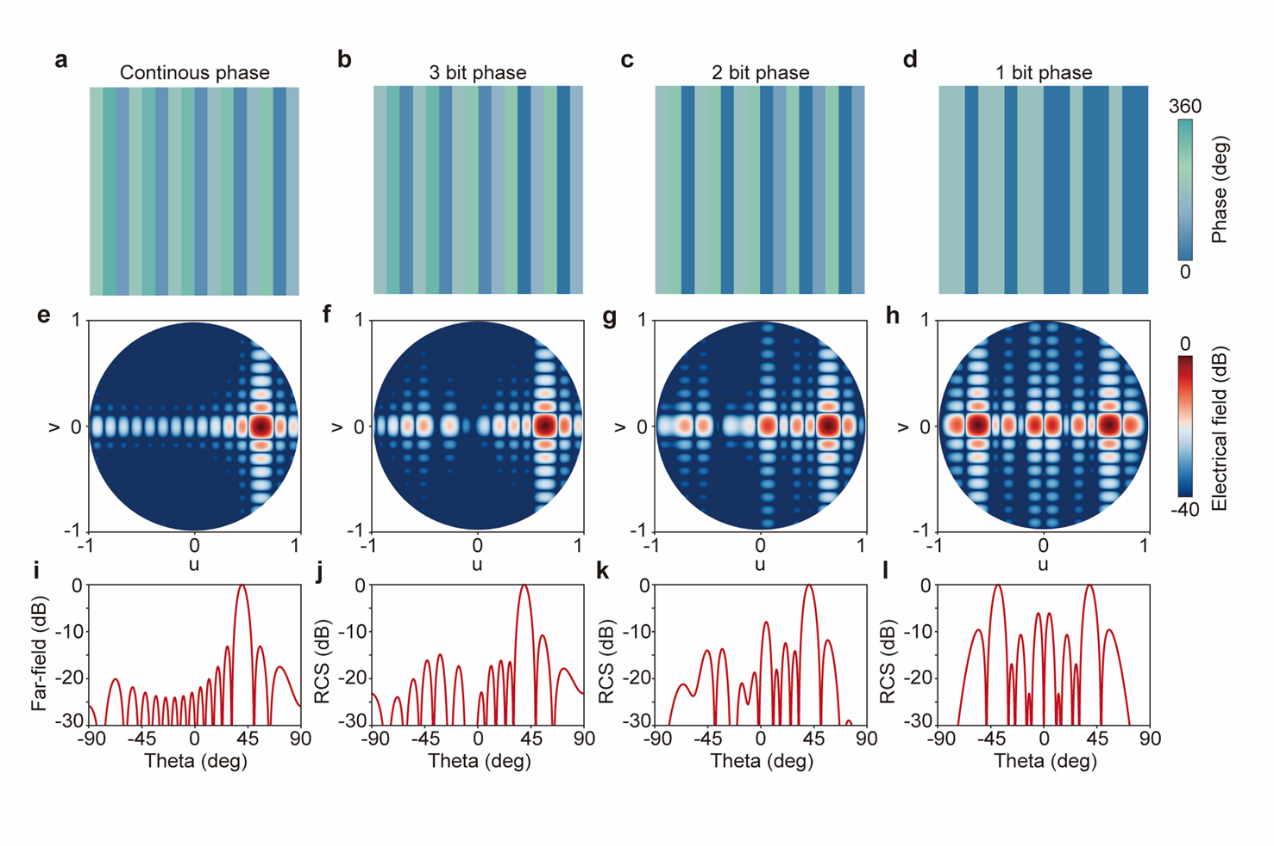


**Figure S6**. 16×16 array steered towards (0°, 45°). Phase distributions for (a) continuous phase, (b) 3-bit quantization, (c) 2-bit quantization, and (d) 1-bit quantization. uv-plane radiation patterns for (e) continuous phase, (f) 3-bit quantization, (g) 2-bit quantization, and (h) 1-bit quantization. Normalized 2D radiation patterns in the φ = 0° plane for (i) continuous phase, (j) 3-bit quantization, (k) 2-bit quantization, and (l) 1-bit quantization.


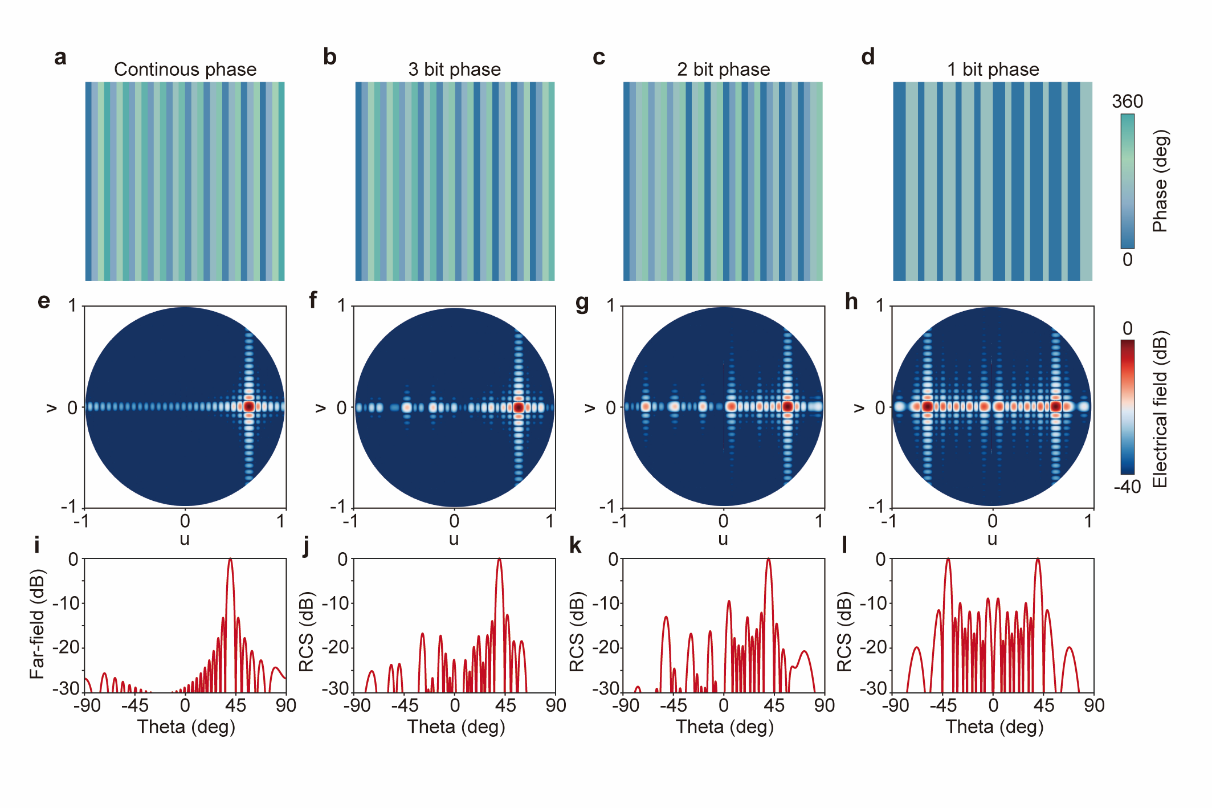


**Figure S7**. 32×32 array steered towards (0°, 45°). Phase distributions for (a) continuous phase, (b) 3-bit quantization, (c) 2-bit quantization, and (d) 1-bit quantization. uv-plane radiation patterns for (e) continuous phase, (f) 3-bit quantization, (g) 2-bit quantization, and (h) 1-bit quantization. Normalized 2D radiation patterns in the φ = 0° plane for (i) continuous phase, (j) 3-bit quantization, (k) 2-bit quantization, and (l) 1-bit quantization.

**Note 4.** **Programmable radiation metasurface enabled solely by geometric phase modulation**

A circularly polarized radiation-type metasurface is designed with an array size of 8 × 8 elements. The initial phase distribution$\text{Φ}_{\text{Ini}}\text{(LHCP)}$ of the co-polarized (LHCP) wave is configured to a spherical wavefront originating from a virtual feed with a focal distance of F = 60 mm, corresponding to a focal-to-aperture ratio F/D = 60/104 = 0.58, as illustrated in Fig. S8(a). When the LHCP initial phase is implemented solely through the in-plane rotation of the radiation patches, an inherent phase term of $\text{Φ}_{\text{Ini}}\text{(RHCP)}\text{=}$–$\text{Φ}_{\text{Ini}}\text{(LHCP)}$ is simultaneously imposed on the RHCP component, as shown in Fig. S8(b). The resulting rotation pattern of the metasurface elements is depicted in Fig. S6(c). Given that the unit cell provides 1-bit phase control for both co- and cross-polarized components (see Figs. 2(i) and 2(j)), the RHCP wave will be deflected in the opposite direction to that of the LHCP beam. This reciprocal scanning behavior adversely affects the cross-polarization level in the (0°, 0°) direction. As demonstrated in Figs. S8(d) and S8(e), the far-field pattern and axial ratio corresponding to the broadside beam (θ = 0°) reveal that the RHCP component is redirected toward the broadside direction, degrading the axial ratio of the array. Even at the center frequency of 10 GHz, the minimum axial ratio barely reaches 3 dB, indicating a poor polarization purity.


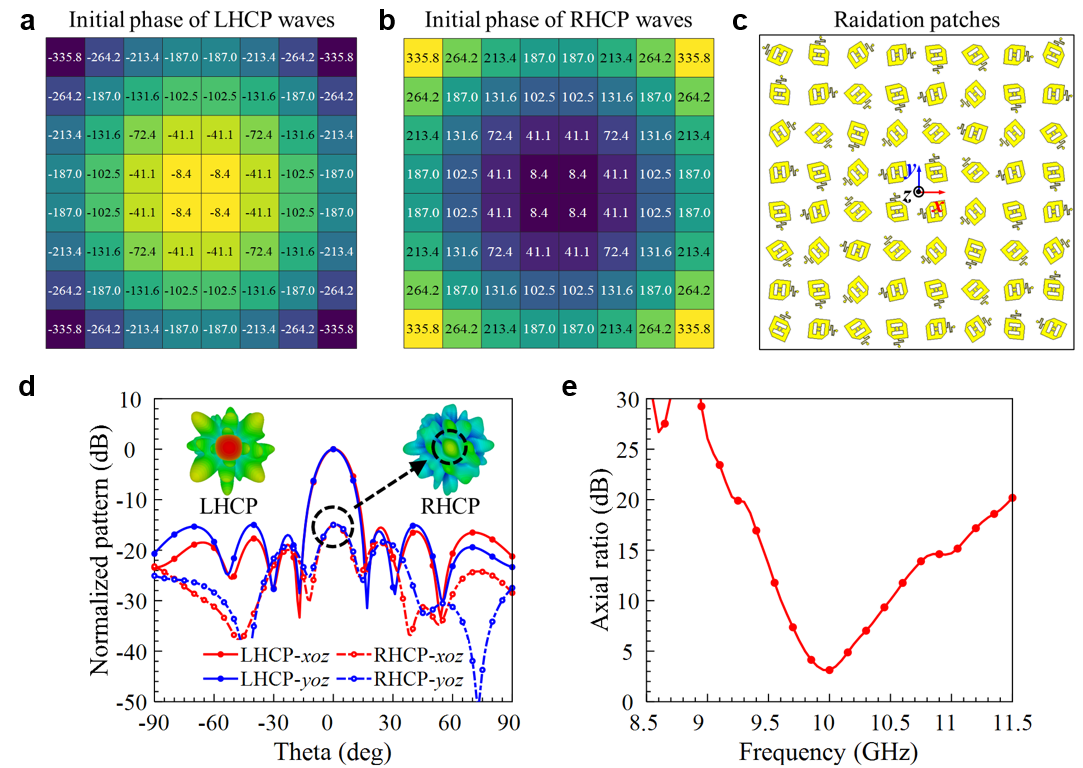


**Figure S8**. Programmable spin-decoupled radiation metasurface enabled by the geometric phase-only method. (a) Initial phase distribution of (a) LHCP waves and (b) RHCP waves, respectively. (c) Radiating patch structure. (d) Normalized pattern (the 3D radiation patterns of both LHCP and RHCP waves are displayed in the insets). (e) AR of the broadside beam.

**Note 5.** **Overall configuration of the proposed PSDM**

The detailed architecture of the DC bias distribution network is depicted in Fig. S9(a). To suppress the cross-polarized component, the propagation phase profile $\text{Φ}_{\text{P}}$ is configured as shown in Fig. S9(b). Correspondingly, a T-shaped power divider network is implemented to feed the metasurface. To streamline the DC biasing process and minimize undesired coupling or interference between the bias lines and RF structures, the bias layout is symmetrically partitioned into four isolated sections, as shown in Fig. S9(c). The 50 Ω and 35.3 Ω microstrip transmission lines are designed with widths of 1.28 mm and 2.18 mm, respectively, to ensure impedance matching. In order to tailor the phase distribution across the sub-arrays, spatial phase delays are introduced by vertically offsetting the primary power division lines. Specifically, an upward shift of length lp in the left branch of the first-stage power divider introduces a phase delay of 𝛷 = lp×*β* in the third 4×4 sub-array, while a downward shift of the same length in the right branch induces a delay of 𝛷 = lp×*β* in the second 4×4 sub-array. The simulated phase constant *β* corresponding to the microstrip line is presented in Fig. S9(d). Thus, the offset parameter can be determined lp = 2.5 mm.


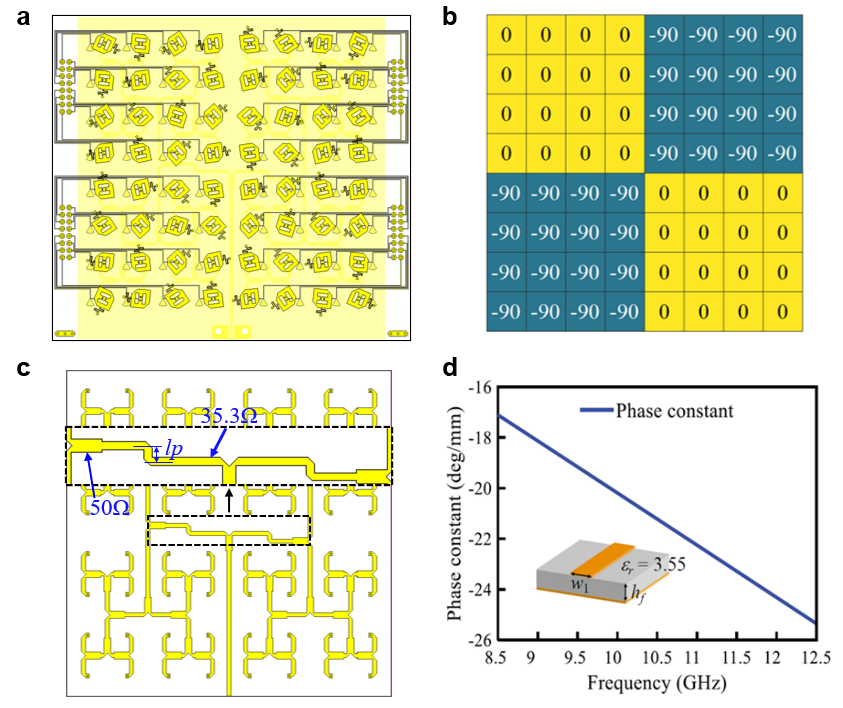


**Figure S9**. Overall configuration of the proposed PSDM. (a) Radiating layer. (b) Prephase distributions of the cross-polarized component. (c) DC bias distribution network. (d) Simulated phase constant β corresponding to the microstrip line.

**Note 6.** **Schematic of the steering-logic board**

The schematic of the steering-logic board is shown in Fig. S10. The input voltage is regulated through two independent buck converter modules: one generates a stable 2.5 V reference voltage, which is applied to the metallic ground plane of the metasurface, so that after experiencing additional structures such as the surrounding feed lines and metasurface feeding network, a sufficient voltage of approximately 1.2 V is provided across the PIN diodes. The other outputs 5 V to power the STM32C8T6 microcontroller unit (MCU). Voltage control is realized via cascaded 74HC595 shift registers, which enable serial-in/parallel-out functionality and support 8-bit individually addressable outputs. The MCU communicates with the shift registers through the SPI protocol, with all clock lines connected to the shared clock bus of the MCU. A 64-bit binary control sequence is transmitted serially along the data line, propagated stage-by-stage through the shift registers, and ultimately translated into either 5 V or 0 V bias voltages for loading onto each PIN diode. This architecture provides precise and scalable control over the transmission states of the programmable metasurface.


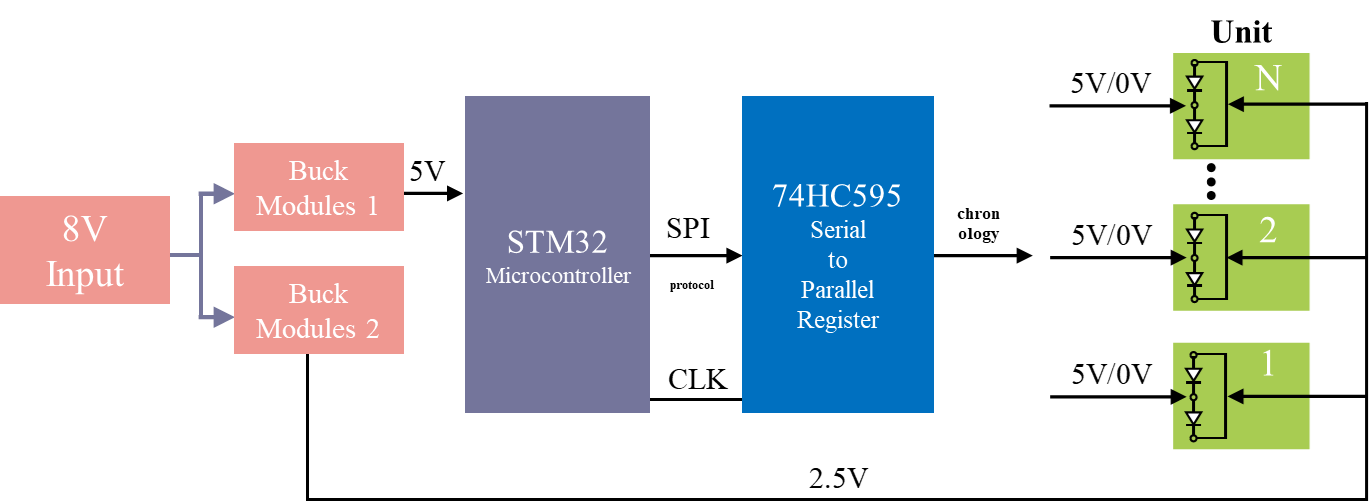


**Figure S10**. Schematic of the steering-logic board.

**Note 7. Radiation patterns of the beam scanning**

Figure S11 illustrates the representative distributions of quantized control codes and their corresponding simulated far-field radiation patterns for beam steering at –45°, –30°, –15°, 0°, 15°, 30°, and 45° in both the *xoz*- and *yoz*-planes at 10 GHz. The results show that well-formed directional pencil beams are accurately generated in the intended directions, demonstrating the effectiveness of the 1-bit programmable coding strategy for beam steering.


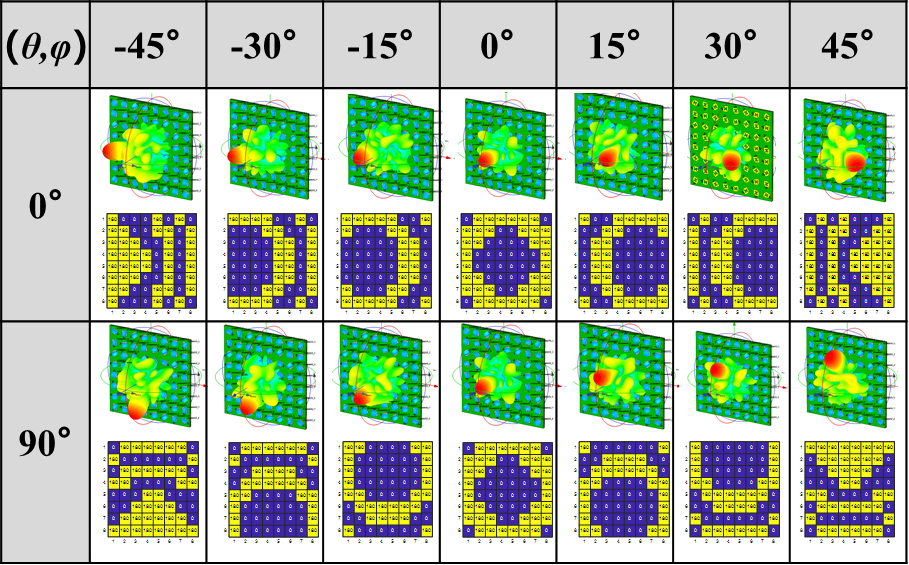


**Figure S11**. Code distributions and simulated far-field radiation patterns for different scanning angles.

**Note 8. Radiation performances of the beam scanning**

Table S1 provides a comprehensive summary of both simulated and measured performance for scanning beams. As the scanning angle increases, the beamwidth broadens gradually, accompanied by a corresponding decrease in peak gain. The measured gain at broadside (0°) reaches 17.1 dBic, and as the beam is steered to ±45° in the xoz- and yoz-planes, the maximum gain reduction is 2.7 dB and 2.2 dB, respectively. The inherent phase quantization of the 1-bit coding scheme introduces phase errors that result in slight deviations between the actual and intended beam directions. The maximum pointing error, observed at –45° in the yoz-plane, is 4.5°, which is significantly smaller than the corresponding 3 dB beamwidth and thus exerts negligible influence on the overall radiation performance of the antenna. In the simulation, the 0° direction is defined with respect to the absolute coordinate system, where the direction perpendicular to the metasurface plane is defined as 0°. However, slight misalignment between the metasurface plane and the horn antenna is unavoidable due to the limited precision of the rotary platform in the experiment. Therefore, an angular calibration was performed by defining the beam maximum of the designed 0° case as the experimental baseline. Consequently, the measured 0° cases appear strictly at 0°, whereas the simulated results may show small offsets. In the yoz-plane, the measured gains at –45°, 15°, and 30° scanning angles are slightly higher than the simulated ones, which is mainly caused by measurement uncertainties such as environmental noise, system calibration, and multipath effects inside the chamber. Nevertheless, both the simulations and measurements consistently confirm the trend of gain reduction with increasing scan angle.

Table S1. Simulated and measured performance of each scanning beam at 10 GHz.


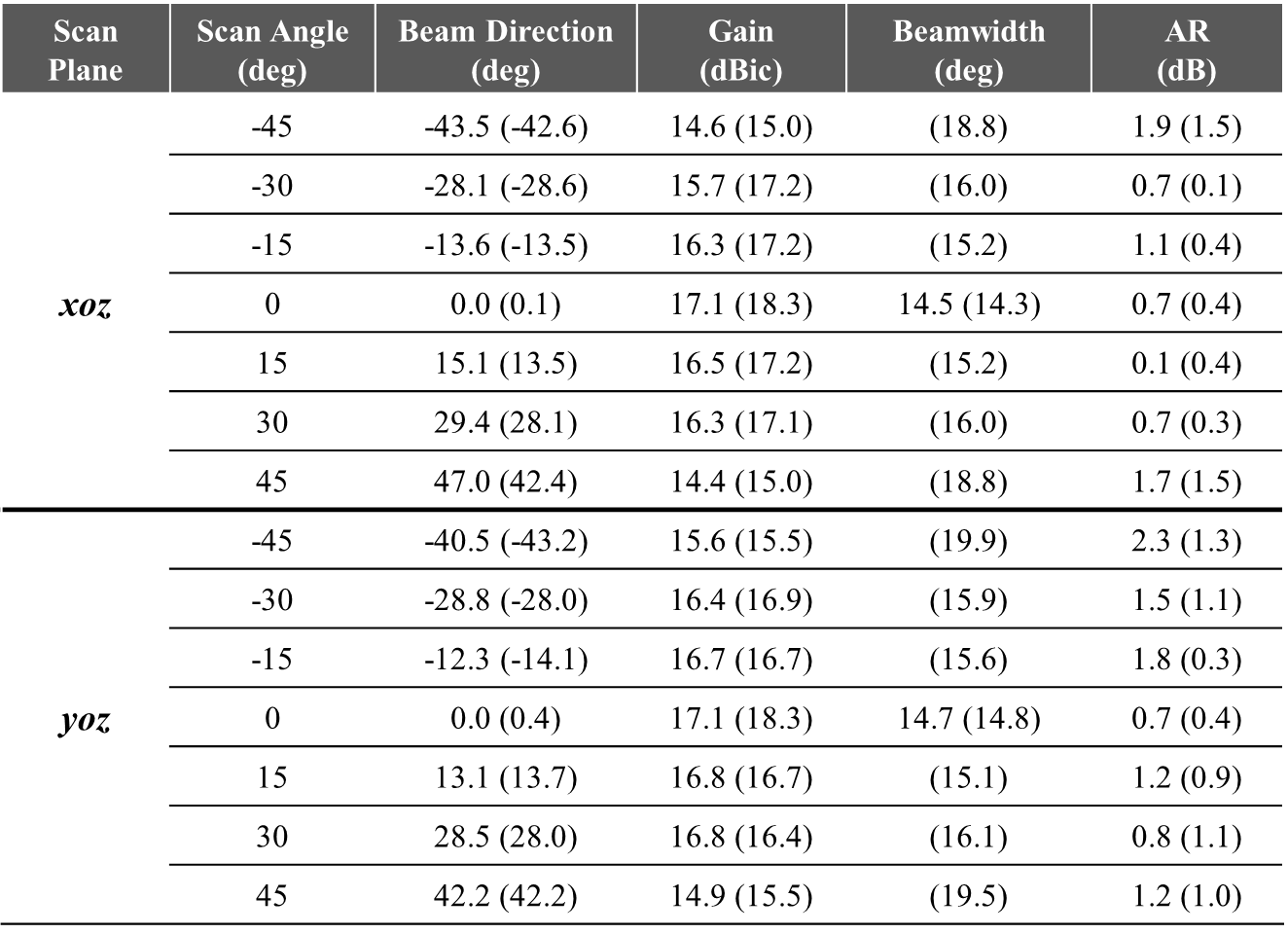


* Values in parentheses represent simulated results, while those outside the parentheses denote measured results.
